# Incorporation of Lipids into Wheat Bran Cellulose/Wheat Gluten Composite Film Improves Its Water Resistance Properties

**DOI:** 10.3390/membranes12010018

**Published:** 2021-12-24

**Authors:** Guanghui Shen, Guoxian Yu, Hejun Wu, Shanshan Li, Xiaoyan Hou, Meiliang Li, Qingye Li, Xingyan Liu, Man Zhou, Anjun Chen, Zhiqing Zhang

**Affiliations:** 1 College of Food Science, Sichuan Agricultural University, Ya’an 625014, China; 13994@sicau.edu.cn (G.S.); ddjll2001@163.com (G.Y.); hejunwu520@163.com (H.W.); lss@sicau.edu.cn (S.L.); liml@sicau.edu.cn (M.L.); qingyeli@sicau.edu.cn (Q.L.); lxy05@126.com (X.L.); zhouman@sicau.edu.cn (M.Z.); anjunc003@163.com (A.C.); 2 Institute of Food Processing and Safety, Sichuan Agricultural University, Ya’an 625014, China; houxiaoyan106@163.com

**Keywords:** hydrophobic composite film, wheat gluten, lipids, beeswax, water barrier properties

## Abstract

This work evaluated the improvement effects of lipids incorporation on water resistance of composite biodegradable film prepared with wheat bran cellulose/wheat gluten (WBC/WG) using an alkaline–ethanol film forming system. Four types of lipids, paraffin wax (PW), beeswax (BW), paraffin oil (PO), and oleic acid (OA), were tested. We found that PW, BW, and PO incorporation at 5–20% improved water vapor permeability (*WVP*) and surface hydrophobicity of prepared films. Particularly, incorporation of 15% BW could best improve the water resistance properties of the film, with the lowest *WVP* of 0.76 × 10^−12^ g/cm·s·Pa and largest water contact angle (*WCA*) of 86.18°. Incorporation of OA led to the decline in moisture barrier properties. SEM images revealed that different lipids incorporation changed the morphology and of the composite film, and cross-sectional morphology indicated BW-incorporated film obtained more uniform and compact structures compared to other films. Moreover, Fourier transform infrared spectra indicated that the incorporation of PW or BW enhanced the molecular interactions between the film components, confirmed by the chemical shift of characteristic peaks at 3277 and 1026 cm^−1^. Differential scanning calorimetry results revealed that incorporation of PW, BW, and PO increased films’ melting point, decomposition temperatures, and enthalpy values. Furthermore, the presence of most lipids decreased tensile strength and elongation at the break of the film. Overall, the composite film containing 15% BW obtained the most promising water resistance performance and acceptable mechanical properties, and it thus most suitable as a hydrophobic biodegradable material for food packaging.

## 1. Introduction

Food packaging is essential for protecting the quality and safety of food products during their storage and distribution. Plastic films are widely used as food packaging materials due to their properties including light weight and water resistance, as well as their low cost. However, most of these plastic films are hardly degradable, and can thus cause serious environmental pollution. Further, some harmful contaminants in the films can migrate into food, which can in turn increase potential risks of foods [1]. Currently, there are growing interests on the development of eco-friendly films with desirable performances that can be applied to produce food packaging materials [2].

Natural polymers, including polysaccharides and proteins, are the best candidates for producing food packaging materials. Not only can these polymers be easily extracted from agricultural by-products, but they also have many advantages, one of which is their degradability in the environment [3]. Wheat gluten (WG) is a by-product from wheat starch manufacturing process; it possesses excellent film-forming and gas barrier properties [4]. WG-based films have good heat-sealing properties and good oxygen and oil resistance properties [5]. However, the WG-based films have poor mechanical and water barrier properties, which have greatly restricted their applications [6]. High contents of water and oxygen inside the food package are the primary factors that can affect quality and shelf-life of food as moisture can change the food quality, increasing chemical reaction and microbial spoilage [7]. Therefore, the mechanical and water barrier properties of WG films ought to be improved to expand their applications.

As a natural polysaccharide polymer with good thermal stability, plant cellulose has been used as a natural reinforcer in food packaging films [8]. Numerous recent studies have shown that incorporating plant-derived celluloses into protein- or starch-based films could improve their mechanical properties [9,10]. These binary-blend matrixes offer the potential to combine different desirable material properties from two individual polymers, to produce optimized films for food packing application. In view of the good film forming and oxygen barrier properties of WG and the excellent mechanical reinforcing effect of wheat bran cellulose (WBC), we developed an alkaline-ethanol film forming system to improve the compatibility of WBC with WG protein, in which we found that the addition of 40% (m/m) WBC could enhance tensile property of WBC/WG binary-blend film, with tensile strength (*TS*) increased from 8.65 to 20.44 MPa [11]. Despite this finding on complementary advantages of WBC and WG, the water barrier properties of resultant binary-blend film were not significantly improved due to the hydrophilic nature of WBC component [12].

Currently, several approaches have been conducted to improve water barrier properties of protein-based films, such as chemical modifications including physical, chemical, and enzymatic treatment [13], and incorporation of hydrophobic agents such as plant essential oils, animal fats, mineral oil, and other lipid materials [6,14,15]. Waxes are generally composed of fatty acids and fatty alcohols. As hydrophobic molecules, some waxes or their compounds have been incorporated (as additives) to improve water barrier properties and to modify hydrophobic properties of protein-based films. These additives exhibited diverse roles in different composite films. One report has shown that candelilla wax can significantly reduce water vapor permeability (WVP) of pea protein isolate-based film, while oleic acid (OA) reduced the water barrier properties and had a plasticizing effect on the film, making it more elastic [14]. Paraffin wax (PW), which is mainly composed of long-chain saturated fatty acids straight-chain hydrocarbons including C16:0 and C18:1, has been shown to best improve water resistance properties of fish gelatin films [16]. Beeswax (BW), a nature wax produced by honeybees has been shown to enhance that of protein-based films but reduce their mechanical properties [17]. BW (10–15%) have also been shown to improve water vapor barriers of gelatin-wax films, but not their mechanical properties [18]. Paraffin oil, also named liquid paraffin, can control the viscosity and stability of emulsion, and exhibited reinforcement effect on the structure compactness and water barrier property of CMC coating [19].

Although previous works have demonstrated the enhancing effects of various types of lipids on water barrier resistance of bio-based film, these published reports focused mainly on the improvement of barrier properties of films by adding lipids (as surfactants) into a pure polysaccharide- or protein-derived emulsion. In fact, the improvement effect of various types of lipids addition on moisture resistance of binary blend film based on plant cellulose and proteins are not well understood. This work thus aimed to investigate the role of lipids in improving the properties, particularly for water resistance properties of WBC/WG binary blend film. In this study, four types of lipids including PW, BW, PO, and OA were incorporated into WBC/WG alkaline-ethanol film forming dispersions to cast various lipid-incorporated ternary blend films. The morphology, thermal stability, and water resistance and mechanical properties of the films were subsequently characterized.

## 2. Materials and Methods

### 2.1. Materials

WG (≥75% protein) was obtained from Fengqiu Huafeng Powder Industry Co., Ltd. (Xinxiang, Henan, China). Wheat bran was purchased from a local market (Ya’an, Sichuan, China). PW (melting temperature range: 53–55 °C) was supplied by Shanghai Hua Shen Rehabilitation Equipment Co., Ltd. (Shanghai, China). BW (melting temperature range: 60–65 °C) was obtained from Tianjin Kaitong Reagent Co., Ltd. (Tianjin, China). PO and OA (analytical pure) were purchased from Chengdu Cologne Reagent Co., Ltd. (Chengdu, Sichuan, China).

### 2.2. Preparation of Wheat Bran Cellulose

Wheat bran cellulose was prepared according to a previously reported method [20]. Briefly, WB was ground using a high-speed grinder; after that, it was washed, dried at 60 °C, and then filtered through an 80-mesh sieve to obtain cellulose powder. The powder was dispersed in distilled water at a ratio of 1:10 (*w*/*v*), and the resultant dispersion was then boiled for 10 min. After its pH was adjusted to approximately 13.0 with 2 mol/L NaOH, the dispersion was heated at 80 °C for 4 h. The sample was subsequently centrifuged at 8000× *g* for 10 min, and the precipitate was washed with distilled water until its pH reached a neutral pH. Afterward, the pH value was adjusted to pH 1.5 using 2 mol/L HCl, and incubated at 60 °C for 2 h in a water bath. The obtained precipitate was filtered and washed with deionized water until its pH became neutral; thereafter, it was dried at 55 °C for 8 h. The dried sample was ground using a superfine grinder and then passed through a 120-mesh sieve to obtain WBC.

### 2.3. Preparation of Film-Forming Dispersions

The film-forming dispersion for WBC/WG films was prepared using an alkaline film-forming system reported in our previous study [11]. In brief, the WG dispersion was prepared by suspending 25.6 g WG in 154 mL of 35% ethanol solution at 45 °C, and the pH was then adjusted to 11.5 using 2.0 mol/L NaOH. Afterward, WG dispersion was heated at 80 °C for 15 min to break disulfide bonds in protein (to denature protein). The WBC dispersion was prepared by dispersing 14.4 g of WBC in 160 mL of deionized water using a high-pressure homogenizer, and was then poured into the prepared WG dispersion. After that, 8.00 g glycerol (as plasticizer) and 0.06 g xanthan gum (as a stabilizer) were mixed at 45 °C and were then added into the WBC/WG dispersion and mixed vigorously. Subsequently, 86 mL of absolute ethanol was added into the above dispersion to a final concentration of 35% under vigorous stirring. The dispersion pH was adjusted to 11.5 and then subjected to a high-pressure homogenizer at 25 MPa for 10 min.

An emulsifier containing monoacylglycerides and Tween-80 at a mass ratio of 2:3 was first melted at 70 °C in a water bath. The melted emulsifier (10%) was then mixed with different concentrations of lipids (5%, 10%, 15%, and 20% (*w*/*w*), dry film basis) to generate transparent lipid solutions, which were further melted at the same temperature. The melted lipid solutions were subsequently mixed with the prepared WBC/WG film-forming dispersion by homogenizing at 13,000 r/min for 15 min at 70 °C (to allow lipids to remain in the liquid state) using a homogenizer (FJ200-SH, Shanghai Specimen Model Factory, Shanghai, China). The obtained film-forming dispersions were eventually vacuum-degassed at −0.09 MPa for 30 min.

### 2.4. Preparation of Films

All films were casted from the above prepared degassed film-forming dispersions in leveled PMMA (polymethyl methacrylate)-coated plates (dimensions: 15 × 15 cm) at 55 °C for 5 h. The films were dried at 25 °C in a desiccator at 51 ± 1% relative humidity (RH) for 48 h [9], and then peeled off and subjected to following characterization in Section 2.5. The WBC/WG films without lipids addition were used as the control.

### 2.5. Characterization of Films

#### 2.5.1. Scanning Electron Microscopy (SEM)

The prepared films were cut into 10 × 10 mm^2^ squares and dried in a desiccator containing silica gel for 2 weeks. After that, the surface and cross-sectional morphology of the films were observed under a scanning electron microscope (Ultra 55, Carl Zeiss AG, Oberkochen, Germany).

#### 2.5.2. Fourier Transform Infrared (FT-IR) Spectroscopy

FT-IR spectra of the films were recorded on a Nicolet IS10 FT-IR spectrometer (Thermo Fisher Scientific, Waltham, MA, USA) equipped with a Smart iTR diamond attenuated total reflectance (ATR) accessory. The spectra data was scanned from 4000 to 650 cm^−1^ at a resolution of 4 cm^−1^ for 32 scans per film.

#### 2.5.3. Differential Scanning Calorimetry (DSC)

DSC curves were recorded using a differential scanning calorimeter (Q200M, TA Instruments, New Castle, DE, USA). The films (approximately 5 mg) were sealed in an aluminum pan and then heated from 25 to 350 °C at a rate of 10 °C/min. An empty pan was used as a blank control.

#### 2.5.4. Determination of Water Solubility

Dried films (dimensions: 20 × 20 mm, dried at 105 °C, weight: *W*1) were shaken in 25 mL of distilled water at 25 °C for 24 h. Afterward, the films were removed from water and then dried to a constant weight at 105 °C. The dried films were weighted to obtain *W*2. Water solubility (*WS*) was calculated from *W*1 and *W*2 by the following equation:

$$WS\;\left(\%\right)=\frac{W1-W2}{W2}\times 100$$

#### 2.5.5. Water Vapor Permeability (*WVP*)

Water vapor permeability (*WVP*, g/cm·s·Pa) of the films was determined using a modified ASTM E96-04 gravimetric method [21]. The prepared films (20 × 20 mm) were sealed in Payne permeability cups containing anhydrous calcium chloride at 25 °C and 81% RH for 7 d, during which they were weighed every 24 h using an analytical balance (±0.0001 g). Water vapor transmission rate (*WVTR*) and *WVP* were calculated according to the following equations:

$$WVTR=\frac{\Delta m}{A\times T}$$

where Δ*m* is the weight gain (g), *A* is the exposed area of the film (cm^2^), and *T* is the time interval (s).

$$WVP=\frac{WVTR\times d}{\Delta P}$$

where *d* is the measured film thickness (cm) and Δ*P* is the water vapor pressure across the film (Pa).

Film thickness was measured at five random positions by a digital micrometer (Zhongtian Experimental Instrument Co., Ltd., Zhengzhou, China) at an accuracy of 1.0 μm.

#### 2.5.6. Water Contact Angle (*WCA*)

*WCA* values on both sides of film were measured using a video-based contact angle measuring device (OCA-H200, Dataphysics Co., Ltd., Filderstadt, Germany). One microliter of distilled water was deposited onto surface of the film (dimensions: 30 × 30 mm), and the droplet images were recorded and used to estimate the *WCA* values. Each measurement was carried out three replicates at random positions on the surface of film.

#### 2.5.7. Mechanical Properties

Tensile strength (*TS*, MPa) and elongation at break (*EAB*, %) of the film were determined at 25 ± 1 °C using a texture analyzer (TA. XT Plus, Stable Micro Systems, London, UK) according to previously published method [22] with minor modifications. The film samples (dimensions: 50 × 10 mm) were fixed on the probe. The cross-head speed was set at 1 mm/s, and the initial distance was set at 30 mm. *TS* and *EAB* values were calculated from the stress-strain curves. The test environment was maintained at 51 ± 1% RH. Six replicates of each sample were performed, and the data were averaged.

### 2.6. Statistical Analysis

All data were expressed as means ± SDs. One-way analysis of variance (ANOVA) and Duncan’s multiple range test were performed using SPSS 27.0 (SPSS, Chicago, IL, USA) at a significant level of *p* = 0.05. Data fitting was carried out on OriginPro 9.1 (OriginLab Corp., Northampton, MA, USA).

## 3. Results and Discussion

### 3.1. Film Morphology

**Figure 1:**
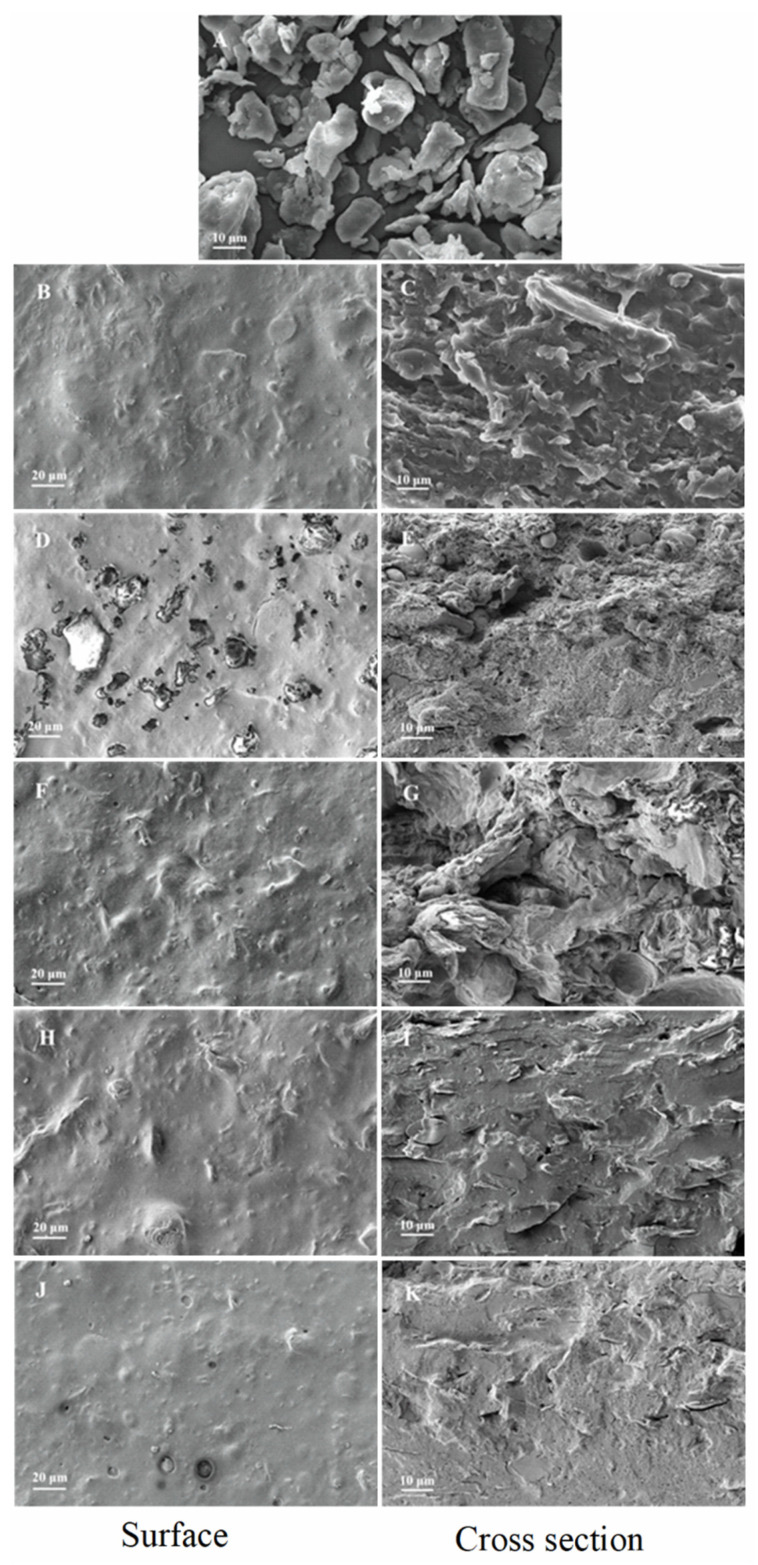
SEM images of wheat bran cellulose (WBC) (×1000 magnification), surface morphology (×500 magnification) and cross section morphology (×1000 magnification) of WBC/WG composite films. WBC (**A**); WBC/WG films (**B**,**C**); paraffin wax–incorporated WBC/WG films (**D**,**E**); beeswax–incorporated WBC/WG films (**F**,**G**); paraffin oil–incorporated WBC/WG films (**H**,**I**); and oleic acid–incorporated WBC/WG films (**J**,**K**).

Figure 1 shows the morphology of native WBC film, the air-side surface, and the cross-section of WBC/WG film containing 15% lipids. The native WBC had a long block-like shape (Figure 1A). The surface of WBC/WG film was inhomogeneous containing a few particles (Figure 1B) of the unincorporated WBC or WBC/WG aggregates. By contrast, the cross-section of the WBC/WG film exhibited a fish scale-like shape and a compact network and was pore free (Figure 1C).

The morphology of lipid-incorporated composite films depended on types of lipids. As illustrated in Figure 1D, numerous lipid aggregate was observed on the air-side surface of PW-incorporated film. Additionally, the cross-section of PW-incorporated films was inhomogeneous, and the structure close to the surface of PMMA side was denser, as can be observed in Figure 1E. The aggregated particles distributed in the top half of the cross-section close to the air-side likely were lipid globules. The roughness of the air-side surface of the PW-incorporated film may be caused by phase separation during the drying process of films and resulted in lipids aggregated at the air-film interface. Smaller aggregated particles on the air-side surface of BW-incorporated film were also observed (Figure 1F). The cross-sectional morphology of BW-incorporated film indicated uniform and compact structures with a few micropores inside the film, and BW particles were tightly embedded in the film (Figure 1G). These observed morphology characteristics suggested that the incorporated BW was uniformly distributed as micro lipid droplets dispersed in the composite film emulsion. Such a phenomenon has been observed in gelatin-beeswax films previously reported by Zhang et al. [18].

On the contrary, incorporation of PO or OA showed slightly effects on morphology of the WBC/WG film. Despite of the obviously particles on the surface of PO-incorporated film (Figure 1H) compared with the control (Figure 1B), its cross-section possessed more ordered structures (Figure 1I). In addition, PO and other components in the cross-section of PO-incorporated film were hardly distinguishable. The cross-sectional morphology of OA-incorporated film, however, showed that the film contained numerous small air bubbles (Figure 1K), which may be generated due to incomplete defoaming during the preparation of film dispersions. It is worth mentioning that when incorporating OA into WBC/WG dispersion, a large amount of air bubbles formed, and only a small amount of which were defoamed. These bubbles may try to escape into air during film drying, in turn causing circular holes with basin shape on the surface of OA-incorporated film (Figure 1J). In the presence of NaOH in film dispersions, OA could convert into sodium oleate, which possesses excellent foaming properties [23] and subsequently contributed to air bubbles. Altogether, we inferred that morphology of the film varies with the physical state and chemical composition of the lipids incorporated.

### 3.2. FT-IR Spectra

**Figure 2:**
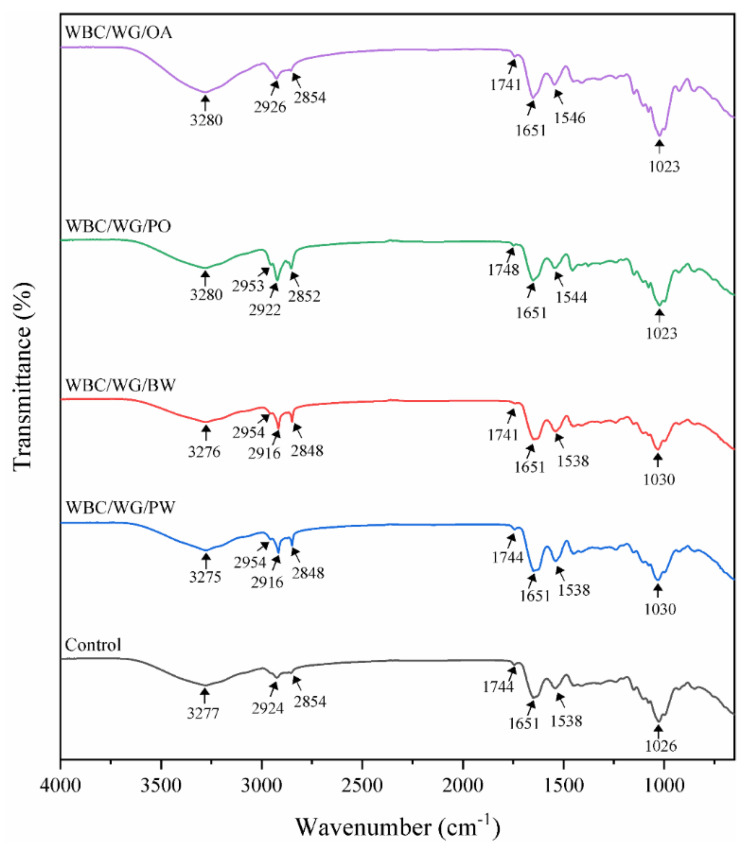
FTIR spectra of composite WBC/WG film incorporated with four types of lipids. WBC/WG: wheat bran cellulose/wheat bran composite film; WBC/WG/PW: paraffin wax–incorporated WBC/WG film; WBC/WG/BW: beeswax–incorporated WBC/WG film; WBC/WG/PO: paraffin oil–incorporated WBC/WG film; WBC/WG/OA: oleic acid–incorporated WBC/WG film.

As presented in Figure 2, FT-IR spectra of the WBC/WG film exhibited the characteristic bands as follows: 3500–3200 cm^−1^ (–OH stretching vibrations), 2800–3000 cm^−1^ (C–H stretching vibrations), 1200–1800 cm^−1^ (carboxylic groups region stretching vibrations), and around 1020–1030 cm^−1^ (C–O stretching vibrations) [24,25]. These bands were slightly changed after the lipids’ incorporation, and their changes were dependent on types of the used lipids. Compared with WBC/WG film, the band at 3277 cm^−1^ of PO- and OA-incorporated films was slightly blue-shifted (to a higher wavenumber) to 3280 cm^−1^, which indicates that incorporation of the liquid lipids (PO and OA) can weaken hydrogen bonding interaction between matrix components [25]. By contrast, incorporation of the two solid lipids (waxes) caused a slight red-shift (to lower wavenumber), indicating the interaction between the components was stronger [26]. Sharp peaks at 2916 and 2848 cm^−1^ due to the asymmetric and symmetric stretching vibrations of aliphatic C-H groups in fatty acid chains, which are the characteristic peaks of most lipids [16], were not observed in the lipid-free WBC/WG film, but were observed in the WBC/WG film containing two solid lipids. Furthermore, a band at 1538 cm^−1^, which is the characteristic band of N–H deformation [27], was shifted to a higher wavenumber upon the incorporation of PO or OA. Additionally, the characteristic band at 1026 cm^−1^ was shifted to 1030 cm^−1^ upon the incorporation of solid lipids, but was shifted to a lower wavenumber of 1023 cm^−1^ when liquid lipids were incorporated into the films. These shifts demonstrate the incorporation of lipids can change the molecular interaction in the composite films. This is in line with a previous report by Zhang et al. [25], which has described that the addition of hydrophobic agents can cause distinct changes to the molecular interaction and in turn causes the characteristic peaks to shift.

### 3.3. Thermal Properties of Film

**Figure 3:**
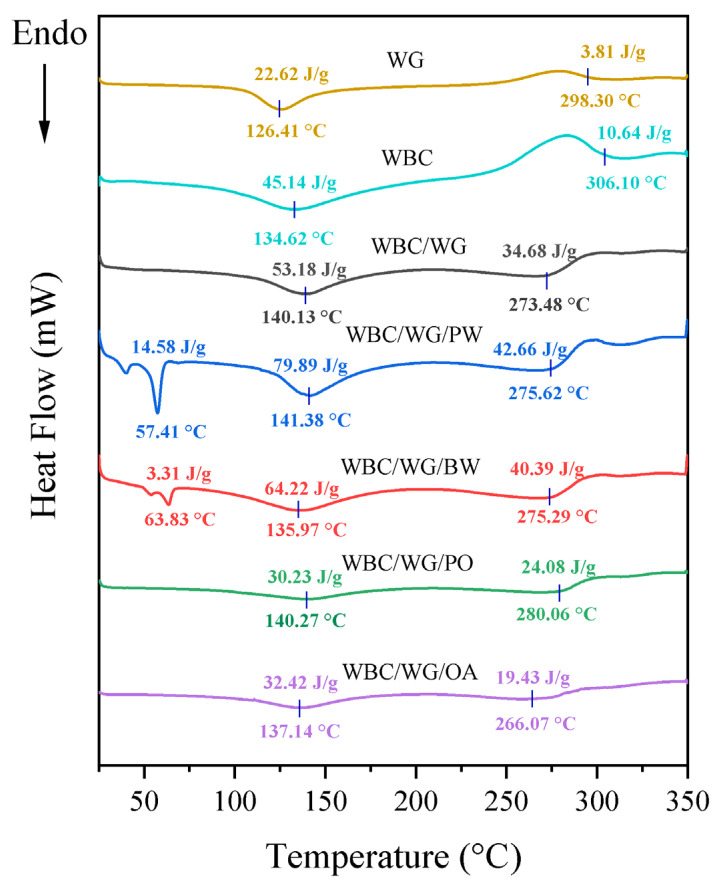
DSC curves of wheat bran (WG), wheat bran cellulose (WBC) and WBC/WG composite films incorporated with four types of lipids. WBC/WG: wheat bran cellulose/wheat bran composite film; WBC/WG/PW: paraffin wax–incorporated WBC/WG film; WBC/WG/BW: beeswax–incorporated WBC/WG film; WBC/WG/PO: paraffin oil–incorporated WBC/WG film; WBC/WG/OA: oleic acid–incorporated WBC/WG film.

The differential scanning calorimetry was particularly used to evaluate the changes in phase transitions of bio-based polymer films during the thermal processing [28,29]. The DSC curves, melting temperature, and enthalpy values of all films are illustrated in Figure 3. The WBC/WG film exhibited two endothermic peaks at 140.13 and 273.48 °C, which may be associated with the combined melting and thermal decomposition of WBC/WG film matrix. The films containing solid lipids exhibited two endothermic peaks at the temperature range of 30–70 °C, which are the melting temperature of the solid lipids [25]. The two endothermic peaks of WBC/WG film were shifted to higher temperatures at 141.38 and 275.62 °C in PW-incorporated film, indicating PW enhanced the thermal stability of the WBC/WG film. On the other hand, the incorporation of BW caused the melting peak at 140.13 °C to shift to a lower temperature at 135.97 °C. It appears that BW, which has a higher melting point than other lipids, may not be homogeneously dispersed in the films during film drying, thereby causing obvious phase transition and separation [25]. The incorporation of PO did not cause the change of the melting temperature, but could increase the decomposition temperature, which indicates PO can enhance the thermal stability of WBC/WG film. Incorporation of OA greatly decreased the temperatures of the two endothermic peaks, causing the film to have a lower thermal stability.

In addition to the DSC curves, enthalpy values (Δ*H*) are another indicator that can indicate the molecular interactions in the amorphous phase and the crystalline structure of the films [30]. As shown in Figure 3, the incorporation of PW, BW, or PO caused the increase of the enthalpy values of the composite films, indicating that these lipids can slightly increase the thermal stability of the films. This is due to that the incorporation of lipids allows strong and compact network structure to be formed [25]. Specifically, the incorporation of lipids (PW, BW, or PO) enhanced the molecular interactions between WBC and WG in the composite film, in turn increasing its thermal stability. By contrast, a large decrease of the enthalpy values of the OA–incorporated film was observed, indicating that the incorporation of OA could decrease the thermal stability of the films. These results indicate that only PW, BW, and PO can improve the thermal stability of WBC/WG films, likely due to the intrinsic physicochemical properties of these lipids [25].

### 3.4. Water-Resistance Properties

#### 3.4.1. Water Solubility

The mechanical characteristics of packaging films are determined by its use [29]. It is believed that lower water solubility (*WS*) of a film is desirable when applied to preserve lower moisture content food inside the food package during storage. As shown in Table 1, the incorporation of lipids decreased WS values of the films. A similar observation caused by the incorporation of waxes has also been reported [18,31]. Comparing all lipids, OA could best reduce the *WS* values of the film. This is most likely due to that OA could distributed more uniformly in film-forming emulsion as compared to other used lipids, and could thus more effectively minimize the contact area between the film and water molecules.

#### 3.4.2. Water Vapor Permeability (*WVP*)

The higher water vapor permeability (*WVP*) is the major drawback of biopolymers films, due to the hydrophilic nature of protein and cellulose matrix [32]. *WVP* values of all composite films are listed in Table 1. The *WVP* values of films containing 15% PW, BW, PO, and OA were lowest with values of about 51.9%, 53.1%, 47.8%, and 32.5%, respectively, and were lower than that of the control (1.63 × 10^−12^ g/cm·s·Pa). PW, BW, PO, and OA are hydrophobic lipids, and could thus effectively repel moisture and reduce hydrophilic interactions; thus, they could significantly decrease the *WVP* values of the WBC/WG composite films. PW has also been incorporated in LDPE/PW composite film and found to lower the *WVP* value of the film [33]. However, as compared with those of the composite films containing 15% lipids, the *WVP* values of the composite films containing 20% lipids were slightly higher. This may be due to that when a higher lipid concentration was used, emulsification may be inadequate, causing the structure of film matrix to be inhomogeneous or destructed (Figure 1). A similar finding has also been previously reported by Zhang et al. [25]. Considering water barrier properties of all films, the film containing PW or BW had significantly (*p* < 0.05) lower *WVP* values than that containing PO or OA. This suggests that solid lipids (PW and BW) could more effectively enhance the water barrier properties of the films than liquid lipids (PO and OA). The different effects of lipids on reducing the *WVP* of the composite films not only are associated with the natures of the lipids, such as physical state, arrangement of chemical bonds, and hydrophobicity, but also are influenced by their interactions with other film’s components [25].

#### 3.4.3. Water Contact Angle (*WCA*)

*WCA* values of all films are shown in Table 1. The *WCA* values on both sides of most films were less than 90°, indicating that the films are hydrophilic. Furthermore, the *WCA* values of the air-side surfaces were lower than those of the PMMA-side surfaces, due to the air-side surfaces being coarser than the PMMA-side surfaces. Moreover, the *WCA* value of the film containing PW, BW, or PO was higher than that of the control. With the increase of lipid concentration, the WCA value of the PMMA-side surface of the film containing PW or BW increased, whereas that of the air-side surface decreased, which may be due to the increase of roughness of the air-side surface in the presence of more lipid (Figure 1). These show that the contact angle and wetting behavior of solid surfaces are affected by surface roughness, inhomogeneity, and particle shape and size. Unlike those of hydrophobic materials, the *WCA* values of hydrophilic materials usually decrease with the increase of surface roughness [34]. The *WCA* value of the PMMA-side surface of the film containing 20% PW increased up to 95.46°, indicating that this surface became more hydrophobic in the presence of 20% PW. In contrast, the *WCA* value of the air-side surface of this film was not significantly changed. The *WCA* values of both sides of the film containing PO increased with increasing lipid concentration. The incorporation of 20% PO caused the *WCA* value of the air-side and the PMMA-side surfaces of the film to increase to 86.30 and 84.84°, respectively, which were 54.71% and 14.82%, respectively, higher than those of the control. These observations show that PW, BW, and PO, which are highly hydrophobic, can effectively block the interactions between hydrophilic groups and water molecules. The decreased *WCA* values of both sides of the PO-incorporated film indicated OA reduces the hydrophobicity of the film.

### 3.5. Mechanical Properties

**Figure 4:**
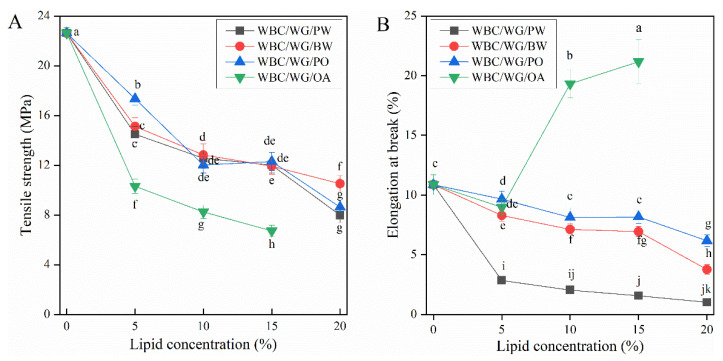
Tensile strength (**A**) and elongation at break (**B**) of WBC/WG composite films incorporated with four types of lipids. WBC/WG/PW: paraffin wax–incorporated WBC/WG film; WBC/WG/BW: beeswax–incorporated WBC/WG film; WBC/WG/PO: paraffin oil–incorporated WBC/WG film; WBC/WG/OA: oleic acid–incorporated WBC/WG film. The values sharing different superscript letters were statistically different at *p* < 0.05.

The tensile strength (*TS*) and elongation at break (*EAB*) are critical mechanical parameter of food packing materials. Strong tensile strength and low elongation are significant to provide the good protection of foods during circulation and storage [29]. Figure 4 shows *TS* and *EAB* values of the prepared films. According to Figure 4A, the incorporation of lipids of all types caused *TS* to significantly decrease from 22.6 MPa to between 9 and 17.33 MPa. The degree of decrease, however, varied with the types and concentrations of lipids. Specifically, among all lipids, the incorporation of OA resulted in the lowest *TS*; and within OA with different concentrations, 15% OA resulted in the lowest *TS* (value = 7.5). Comparing all lipids at different concentrations, the higher the lipid concentration, the lower the *TS* value, which is in line with many previously reported studies [16,18]. These results can be explained by lipids having a non-polymeric nature that limits their cohesive film-forming capacity [15], which in turn causes the film to have heterogeneous structure [35].

Furthermore, as shown in Figure 4B, the incorporation of PW, BW, or PO caused the *EAB* to significantly decrease. This decrease may be caused by solid particles that can cause the formation of rigid dispersed phase in the film, in turn leading to decreased flexibility of the film [36]. In contrast, the incorporation of OA increased the *EAB* value, suggesting that OA has a strong plasticizing effect on the films, causing the network structure of the film to be discontinuous [37]. Similar results have also been previously observed [3,16].

## 4. Conclusions

In this paper, an alkaline-ethanol film forming system was successfully applied to develop hydrophobic WBC/WG blended films by incorporating four types of lipids. The effects of lipids addition on the water barrier properties and morphology of the blended films are dependent upon the physical state and chemical constituents of lipids. The incorporation of two solid lipids (PW and BW) showed more effectively improvement on water-resistance properties of WBC/WG films as compared to two liquid lipids (PO and OA). However, OA incorporation decreased the mechanical properties of the films, as indicated by their lower *TS* and higher *EAB* values. Moreover, the FTIR spectra revealed that the incorporation of PW and BW enhanced the molecular interaction between the film components, while the incorporation of PO and OA attenuated it. The DSC results indicated that the incorporation of lipids changed the inter-molecular interaction among the components of the films, thus their thermal properties. The incorporation of 15% BW could best improve the water resistance properties of WBC/WG film, lowering its *WVP* value (to 0.76 × 10^−12^ g/cm·s·Pa) and increase its *WCA* (to 86.18° on the PMMA-side surface), and improve its mechanical properties. Collectively, the incorporation of BW could best improve the water resistance properties of WBC/WG hydrophobic films for food packaging. Further research should focus on developing BW/WBC/WG-based films with good antibacterial properties by adding natural antimicrobial substances into films dispersion, and further evaluation of potential fresh-keeping effects of films for food packaging is needed.

## Figures and Tables

**Table 1:** Effect of incorporation of lipids on water solubility (*WS*), water vapor permeability (*WVP*) and water contact angle (*WCA*) of WBC/WG composite films.

Lipid Type	Lipid Concentration (%)	*WS* (%)	*WVP* (10^−12^ g/cm·s·Pa)	*WCA* (°)	
				Air-Side	PMMA-Side
Control	0	22.59 ± 0.26 ^a^	1.63 ± 0.01 ^a^	55.78 ± 3.27 ^e^	73.89 ± 3.00 ^e^
PW	5	21.64 ± 0.18 ^b^	1.14 ± 0.08 ^def^	68.86 ± 3.56 ^cd^	73.60 ± 2.79 ^e^
	10	21.06 ± 0.36 ^bcd^	1.02 ± 0.03 ^gh^	56.60 ± 4.83 ^e^	74.28 ± 2.92 ^e^
	15	19.91 ± 0.45 ^ef^	0.78 ± 0.06 ^ij^	56.17 ± 3.60 ^e^	84.88 ± 5.31 ^b^
	20	18.88 ± 0.52 ^gh^	1.01 ± 0.05 ^h^	54.81 ± 0.72 ^e^	95.46 ± 2.01 ^a^
BW	5	21.31 ± 0.44 ^bcd^	1.23 ± 0.03 ^cd^	73.70 ± 4.06 ^bc^	75.34 ± 2.87 ^de^
	10	20.80 ± 0.32 ^cd^	1.12 ± 0.05 ^ef^	67.50 ± 2.91 ^cd^	80.58 ± 3.54 ^bcd^
	15	19.82 ± 0.62 ^efg^	0.76 ± 0.02 ^j^	66.68 ± 2.46 ^d^	86.18 ± 1.20 ^b^
	20	18.50 ± 0.25 ^h^	1.02 ± 0.06 ^gh^	57.96 ± 0.65 ^e^	85.62 ± 2.53 ^b^
PO	5	21.54 ± 0.34 ^bc^	1.39 ± 0.04 ^b^	66.95 ± 3.74 ^d^	75.02 ± 0.55 ^de^
	10	20.46 ± 0.48 ^def^	1.17 ± 0.04 ^def^	76.68 ± 3.32 ^b^	77.72 ± 1.60 ^cde^
	15	19.77 ± 0.57 ^efg^	0.85 ± 0.03 ^i^	91.21 ± 3.50 ^a^	81.90 ± 4.53 ^bc^
	20	19.29 ± 0.26 ^gh^	1.10 ± 0.04 ^fg^	86.30 ± 4.94 ^a^	84.84 ± 3.31 ^b^
OA	5	20.76 ± 0.39 ^bc^	1.28 ± 0.05 ^c^	51.43 ± 3.87 ^ef^	64.87 ± 1.09 ^f^
	10	19.44 ± 0.55 ^fgh^	1.20 ± 0.10 ^cde^	46.80 ± 2.39 ^fg^	63.99 ± 0.89 ^f^
	15	18.82 ± 0.60 ^gh^	1.09 ± 0.02 ^fgh^	41.04 ± 1.44 ^g^	62.56 ± 4.29 ^f^
	20	n.a.	n.a.	n.a.	n.a.

The values sharing different superscript letters in the same column were statistically different at *p* < 0.05 by Duncan’s multiple range test; n.a. not analyzed. Film incorporated 20% OA not evaluated because hardly peel off.

## Data Availability

Not applicable.
